# Artificial intelligence in interventional radiology: state of the art

**DOI:** 10.1186/s41747-024-00452-2

**Published:** 2024-05-02

**Authors:** Pierluigi Glielmo, Stefano Fusco, Salvatore Gitto, Giulia Zantonelli, Domenico Albano, Carmelo Messina, Luca Maria Sconfienza, Giovanni Mauri

**Affiliations:** 1 Dipartimento di Scienze Biomediche per la Salute, Università degli Studi di Milano, Via Mangiagalli, 31, 20133 Milan, Italy; 2 IRCCS Istituto Ortopedico Galeazzi, Via Cristina Belgioioso, 173, 20157 Milan, Italy; 3 Dipartimento di Scienze Biomediche, Chirurgiche ed Odontoiatriche, Università degli Studi di Milano, Via della Commenda, 10, 20122 Milan, Italy; 4 Divisione di Radiologia Interventistica, IEO, IRCCS Istituto Europeo di Oncologia, Milan, Italy

**Keywords:** Artificial intelligence, Deep learning, Machine learning, Neural networks (computer), Radiology (interventional)

## Abstract

Artificial intelligence (AI) has demonstrated great potential in a wide variety of applications in interventional radiology (IR). Support for decision-making and outcome prediction, new functions and improvements in fluoroscopy, ultrasound, computed tomography, and magnetic resonance imaging, specifically in the field of IR, have all been investigated. Furthermore, AI represents a significant boost for fusion imaging and simulated reality, robotics, touchless software interactions, and virtual biopsy. The procedural nature, heterogeneity, and lack of standardisation slow down the process of adoption of AI in IR. Research in AI is in its early stages as current literature is based on pilot or proof of concept studies. The full range of possibilities is yet to be explored.

**Relevance statement** Exploring AI’s transformative potential, this article assesses its current applications and challenges in IR, offering insights into decision support and outcome prediction, imaging enhancements, robotics, and touchless interactions, shaping the future of patient care.

**Key points**

• AI adoption in IR is more complex compared to diagnostic radiology.

• Current literature about AI in IR is in its early stages.

• AI has the potential to revolutionise every aspect of IR.

**Graphical Abstract:**

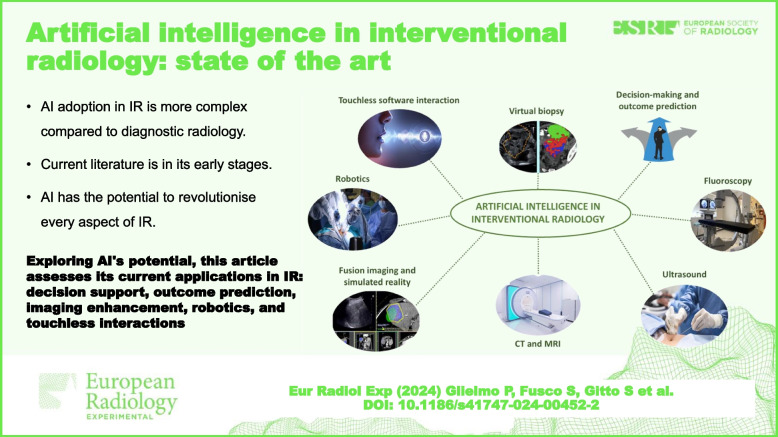

## Background

Perspectives in artificial intelligence (AI) differ and are more complex for interventional radiology (IR) than for diagnostic radiology because IR encompasses diagnostic imaging, imaging guidance, and early imaging evaluation as well as therapeutic tools [1].

Whilst diagnostic radiology is largely based on data acquired in a standardised format, IR, due to its procedural nature, relies on mostly unstructured data. Nevertheless, preprocedural, procedural, and postprocedural imaging constitutes a sizable dataset when compared to other specialties in medicine. In addition, machine learning (ML) and data augmentation techniques reduce the dataset size required for effective training. ML techniques exploit expedients such as in supervised learning (where models learn from labelled examples), few-shot learning (capable of generalising effectively from minimal examples per category), and transfer learning (leveraging knowledge from one task to improve performance on another). Data augmentation techniques enable the creation of synthetic training examples through the transformation of original data (*e.g.,* elastic transformations, affine image transformations, pixel-level transformations) or from the generation of artificial data. Researchers have developed generative AI algorithms that generate artificial radiological images for training. Similar techniques could be adapted for IR obtaining more representative and extensive training data [1, 2].

Another factor that contributes to slowing down the process of adoption of AI in IR is the heterogeneous nature of this subspecialty. IR provides mini-invasive solutions for many different pathologies across multiple organ systems. Intraprocedural imaging, be it ultrasound (US), fluoroscopy, or even computed tomography (CT) or magnetic resonance imaging, can be heavily operator dependent as is the choice of the preferred percutaneous/intravascular approach, guidance, and different devices. This lack of standardisation poses challenges in creating adequate datasets for training and implies the need for flexibility of AI to a number of different situations and options for the same task. Furthermore, being a relatively young technology-based subspecialty, IR is constantly evolving which exacerbates issues related to its inner heterogeneity.

AI in IR is still in its early stages. Much of the literature relies on preliminary and hypothetical use cases. That being said, AI has the potential to improve and transform every aspect of IR. Acknowledging that every improvement of AI in diagnostic radiology affects IR more or less directly, in the following paragraphs, we cover the most promising AI applications present in literature specifically regarding the field of IR. These applications can be divided into the following areas of improvement: decision-making and outcome prediction, fluoroscopy, US, CT, MRI, fusion imaging and simulated reality, robotics, touchless software interaction, and virtual biopsy, as synthesised in Table 1.

**Table 1:** Fields of application of artificial intelligence in interventional radiology

Field of application	Main results available and perspectives	Literature references
Decision-making and outcome prediction	- Morshid et al. developed a model predicting treatment response to TACE in hepatocellular carcinoma. - AI models by Sinha et al. accurately predict pneumothorax and length of stay after procedures. - Daye et al. predict local tumour progression and survival in adrenal metastases. - AI outperformed traditional radiological biomarkers in predicting stroke treatment outcomes. - Nielsen et al. evaluate DL model for objective TICI score in stroke treatments.	[3–14]
Fluoroscopy	- Yang et al. proposed vessel segmentation in coronary angiography using DL models. - Ambrosini et al. introduced automatic catheter segmentation with U-Net. - Gao et al. reduced motion artefacts in subtraction angiography with AI. - AI-equipped fluoroscopy units reduced radiation exposure during endoscopic procedures.	[15–20]
Ultrasound	- Mwikirize et al. improved needle localisation and placement accuracy in ultrasound-guided procedures.	[21, 22]
CT and MRI	- DL techniques enhanced segmentation, registration, and tumour coverage evaluation in thermal ablation. - AI generated synthetic CT images from cone-beam CT, aiding image guidance.	[23–33]
Fusion imaging and simulated reality	- AI is set to facilitate multimodality image fusion as proposed in other fields. - Auloge et al. demonstrated the efficacy of AI-guided percutaneous vertebroplasty, showing comparable accuracy to standard fluoroscopy with reduced fluoroscopy time	[34, 35]
Robotics	- AI helped in handling multimodal data generated in robotic sensing applications.	[36–38]
Touchless software interaction	- Schwarz et al. used AI to improve recognition rates of body gestures.	[39–42]
Virtual biopsy	- Barros et al. developed an AI model for digital mammography, achieving high accuracy in classifying ductal carcinoma *in situ*, invasive carcinomas, and benign lesions.	[43]

*CT* Computed tomography, *MRI* Magnetic resonance imaging

## Decision-making and outcome prediction

AI support in decision-making concerns a great variety of fields other than IR and other specialties in medicine. Interventional radiologists use clinical information and image interpretation for diagnosis and treatment often relying on multidisciplinary boards to improve patient care due to the interdisciplinarity of IR. Traditionally, clinical risk calculators have been developed using scoring systems or linear models validated on a limited patient sample. ML offers the potential to uncover nonlinear associations amongst the input variables missed by these older models. It could incorporate all available data, along with radiomic information, to perform descriptive analysis, assess risks, and make predictions to help tailor the management of a specific patient [3].

In interventional oncology, many AI applications focus on predicting the response of hepatocellular carcinoma to transarterial chemoembolisation (TACE) [4−6]. Up to 60% of patients with hepatocellular carcinoma who undergo TACE do not benefit from it despite multiple sessions. Patient selection guidelines for TACE are based on the Barcelona Clinic liver cancer—BCLC staging system [7]. Higher arterial enhancement and grey-level co-occurrence matrix, lower homogeneity, and smaller tumour size at pretherapeutic dynamic CT texture analysis were shown to be significant predictors of complete response after TACE [8]. However, the accuracy of this method is limited based on traditional statistics. Morshid et al. [4] developed a predictive model by extracting image texture features from neural network-based segmentation of hepatocellular carcinoma lesions and the background liver in 105 patients. The accuracy rate for distinguishing TACE-susceptible *versus* TACE-refractory cases was 74.2%, surpassing the predictive capability of the Barcelona Clinic liver cancer staging system alone (62.9%). Another study predicted TACE treatment response by combining clinical patient data and baseline MRI [9].

Sinha et al. [3] built and evaluated their AI models on large national datasets and achieved excellent predictions regarding two different outcomes in two different clinical settings: iatrogenic pneumothorax after CT-guided transthoracic biopsy and occurrence of length of stay > 3 days after uterine artery embolisation. Area under the receiver operating characteristic curve was 0.913 for the transthoracic biopsy model and 0.879 for the uterine artery embolisation model. All model input features were available before hospital admission.

In another study, a ML algorithm, the model for end-stage liver disease—MELD score, and Child–Pugh score were compared for predicting 30-day mortality following transjugular intrahepatic portosystemic shunt—TIPS. Model for end-stage liver disease and Child–Pugh are popular tools to predict outcomes in patients with cirrhosis, but they are not specifically designed for patients with transjugular intrahepatic portosystemic shunt. However, they performed better than AI that was still able to make predictions out of mere demographic factors and medical comorbidities, data that are absent in these scores [10].

In a pilot retrospective study, Daye et al. [11] used AI to predict local tumour progression and overall survival in 21 patients with adrenal metastases treated with percutaneous thermal ablation. The AI software had an accuracy of 0.93 in predicting local tumour response and overall survival when clinical data were combined with features extracted from pretreatment contrast-enhanced CT.

In another study [12], AI outperformed traditional radiological biomarkers from CT angiography for good reperfusion and functional outcome prediction after endovascular treatment in acute ischemic stroke patients on a registry dataset with 1,301 patients. The predictive value was overall relatively low. Similarly, Hofmeister et al. [13] obtained information on the success of different endovascular treatments based on non-contrast CT in a prospective validation cohort of 47 patients. A small subset of radiomic features was predictive of first-attempt recanalisation with thromboaspiration (area under the receiver operating characteristic curve = 0.88). The same subset also predicted the overall number of passages required for successful recanalisation.

Mechanical thrombectomy success in acute ischemic stroke is commonly assessed by the thrombolysis in cerebral infarction (TICI) score, assigned by visual inspection of digital subtraction angiography during the intervention. Digital subtraction angiography interpretation and subsequent TICI scoring is highly observer dependent. Application of AI in this setting has been investigated. Digital subtraction angiography image data are rarely used in AI due to the complex nature of angiographic runs. Nielsen et al. [14] evaluated the general suitability of a deep learning (DL) model at producing an objective TICI score in case of occlusion of the M1 segment of the middle cerebral artery.

## Fluoroscopy

AI has proved its utility in enhancing performance and diagnostic power and in facilitating the interpretation of fluoroscopic imaging.

Although major vessels have standard views for angiographic acquisition, the angiographic characteristics are influenced by clinical settings, such as view angle, magnification ratio, use of contrast media, and imaging system [44]. Most of the presented models based on angiographic images have the advantage that image preprocessing steps were minimised or cancelled because they are seamlessly integrated into the DL model.

Yang et al. [15] proposed a robust method for major vessels segmentation on coronary angiography using four DL models constructed on the basis of U-Net architecture. This could be a valuable tool for target vessel identification and for easily understanding the tree structure of regional vasculature.

Segmentation and extraction of catheter and guidewire from fluoroscopic images will aid in virtual road mapping of the vasculature from pre-operative imaging. Segmentation methods for electrophysiology electrodes and catheter have been proposed [16, 17]. Electrodes are clearly visible in two-dimensional x-ray images and this specific feature facilitates their segmentation. Ambrosini et al. [18] introduced a fully automatic approach based on the U-Net model that can be run in real time for segmentation of catheter with no specific features.

Due to the spatial inconsistency between mask image (no contrast agent) and live image (with contrast agent) caused by inevitable and complex patient motion, subtraction angiography usually contains motion artefacts and the vessels are blurred, a phenomenon known as inter-scan motion [44]. Numerous image coregistration algorithms have been proposed to reduce motion artefacts, but they are computationally intensive and have not had widespread adoption [45]. AI demonstrated better performances than the compared registration algorithms. In particular, Gao et al. [19] trained a residual dense block on single live images fed into the generator and satisfactorily subtracted images as output. This resulted in subtraction images generated without the preliminary non-contrast acquisition, avoiding the issue of translational motion entirely and reducing the radiation dose [19].

Radiation exposure to the operator remains a relevant issue in IR. Whilst its relevance has diminished in diagnostic radiology with the emergence of radiation-free imaging modalities and the widespread use of CT, which allows a safe distance from the radiation source, interventional radiologists continue to rely on nearby x-rays.

Radiation exposure to both the operator and the patient has been significantly reduced using an AI-equipped fluoroscopy unit with ultrafast collimation during endoscopy [20]. It is easy to imagine its adaptability to IR. During an endoscopic procedure requiring fluoroscopy, the endoscopist is usually focused only on a small region of the displayed field of view that correlates with procedural activities such as the movement of a guidewire or a catheter. The larger area around the region of interest (ROI) receives much less attention but is needed for reference and orientation purposes. With the present technology, the larger area outside the ROI is exposed to the same radiation dosage as the small ROI. The AI-equipped fluoroscopy system can minimise radiation exposure via a secondary collimator by constantly adjusting the shutter’s lead blade orientation to block radiation to the area outside of the ROI for a majority of image frames and overlying the real-time ROI images over a full field of view image acquired a few frames before. Image outside of the ROI aids only in the orientation, and this effect is not perceptible to the operator. Although the ROI is automatically targeted using AI, there is also an optional provision for manual control by the operator [20].

## Ultrasound

Accurate needle placement is crucial in IR procedures aiming at tissue sampling. Needle localisation during US-guided manoeuvres is not always optimal because of lower detection with steep needle-probe angles, deep insertions, reflective signal losses, hyperechoic surrounding tissues, and intrinsic needle visibility [46]. Furthermore, current US systems are not specifically designed for IR and are limited to the diagnostic aspects. Hardware-based approaches for improving needle shaft and tip localisation, for example, external trackers and specialised needles/probes, exist [47, 48]. However, image processing-based methods that do not require additional hardware are easier to adapt in the standard clinical workflow.

Mwikirize et al. [21] used a faster region-based convolutional neural network (Faster R-CNN) to improve two-dimensional US-guided needle insertion. A Faster R-CNN is translational invariant, allowing needles of various sizes to be inserted at different depths and insertion angles, and the detector will perform accurately regardless of the needle’s geometrical transformation. The system allows automatic detection of needle insertion side, estimation of the needle insertion trajectory, and facilitating automatic localisation of the tip. It achieved a precision of 99.6%, recall of 99.8%, and an F1 score of 0.99 on scans collected over a bovine/porcine lumbosacral spine phantom. Accurate tip localisation is obtained even in cases where, due to needle discontinuity, various regions of the needle may be detected separately but this applies only to non-bending needles.

The shortage of high-quality training data from US-guided interventions is particularly pronounced when compared to other imaging modalities. US is inherently operator dependent and susceptible to artefact. Furthermore, the manual annotation of images is more challenging and time-consuming. To address these problems, Arapi et al. [22] employed synthetic US data generated from CT and MRI to train a DL detection algorithm. They validated their model for the localisation of needle tip and target anatomy on real *in vitro* US images, showing promising results for this data generation approach.

### CT and MRI

The efficacy of thermal ablation in treating tumours is linked to achieving complete tumour coverage with minimal ablative margin, ideally at least 5 mm, enhancing local tumour control. Manual segmentation and registration of tumour and ablation zones invariably introduce operator bias in ablative margin analysis and are time-consuming. The registration in particular is challenging due to errors induced by breathing motion and heating-related tissue deformation. Current methodologies lack intra-procedural accuracy, posing limitations in assessing ablative margin and tissue contraction. Several retrospective studies have employed DL to address these difficulties, demonstrating its utility in achieving deformable image registration and auto-segmentation [23–25]. The COVER-ALL randomised controlled trial investigated a novel AI-based intra-procedural approach to optimise tumour coverage and minimise non-target tissue ablation, potentially elevating liver ablation efficacy [26]. Similarly, a separate study [27] demonstrates the effectiveness of DL in segmenting Lipiodol on cone-beam CT during TACE, outperforming conventional methods. This would allow physicians to feel comfortable relying heavily on cone-beam CT imaging and using obtained cone-beam CT data to make predictive inferences about treatment success and even patient outcome.

Regarding cone-beam CT, DL techniques have been successfully used to generate a synthetic CT image from cone-beam CT imaging [28, 29] overcoming the limitations in image contrast compared to multi-detector CT and enhancing a frequently used image guidance system in the IR suite.

Creating synthetic contrast-enhanced CT images has been proposed in diagnostic radiology [30, 31] to reduce usage of iodinated contrast agents. Pinnock et al. proposed a first study on synthetic contrast-enhanced CT in IR, which poses challenges such as organ displacement and needle insertion [32].

MRI-guided interventions are not widespread performed in IR, and most of the time, MRI use is limited to bioptic procedures or fusion imaging [49]. Needle placement is crucial even in these cases. A group of researchers applied three-dimensional CNNs to create a more sophisticated and automatic needle localisation system for MRI-guided transperineal prostate biopsies. Although some of their results were not statistically significant, this group demonstrated a potential for ML applications to improve needle segmentation and localisation with MRI assistance in a clinical setting [33].

### Fusion imaging and simulated reality

Multimodality image fusion is increasingly used in IR and in a variety of clinical situations [50–56]. It allows the generation of a composite image from multiple input images containing complementary information of the same anatomical site for vascular and non-vascular procedures. Pixel level image fusion algorithms are at the base of this technology. By integrating the information contained in multiple images of the same scene into one composite image, pixel level image fusion is recognised as having high significance in a variety of fields. DL-based image fusion is currently in its early stages; however, DL-based image fusion methods have been proposed for other fields such as digital photography and multimodality imaging too, showing advantages over conventional methods and huge potential for future improvement [34].

Simulated reality, along with AI and robotics, represents some of the most exciting technology advancements in the future of medicine and particularly in radiology. Virtual reality and augmented reality (AR) provide stereoscopic and three-dimensional immersion of a simulated object. Virtual reality simulates a virtual environment whilst AR overlays simulated objects into the real-world background [57]. This technology can be used to display volumetric medical images, such as CT and MRI allowing for a more accurate representation of the three-dimensional nature of anatomical structures, thereby being beneficial in diagnosis, education, and interventional procedures.

Interacting with volumetric images in a virtual space with a stereoscopic view has several advantages over the conventional monoscopic two-dimensional slices on a flat panel as perception of depth and distance. Virtual three-dimensional anatomy/trajectory is overlaid onto visual surface anatomy using a variety of technologies to create a fused real-time AR image. The technique permits accurate visual navigation, theoretically without need for fluoroscopy.

Many studies have already utilised simulated reality in IR procedures [58–61]. Fritz et al. [62, 63] employed AR for a variety of IR procedures performed on cadavers, including spinal injection and MRI-guided vertebroplasty. Solbiati et al. [64] reported the first *in vivo* study of an AR system for the guidance of percutaneous interventional oncology procedures. Recently, Albano et al. [65] performed bone biopsies guided by AR in eight patients with 100% technical success.

The primary advantages secured by utilising AI in this setting include automated landmark recognition, compensation for motion artefact, and generation/validation of a safe needle trajectory.

Auloge et al. [35] conducted a 20-patient randomised clinical study to test the efficacy of percutaneous vertebroplasty for patients with vertebral compression fractures. Patients were randomised to two groups: procedures performed with standard fluoroscopy and procedures augmented with AI guidance. Following cone beam CT acquisition of the target volume, the AI software automatically recognises osseous landmarks, numerically identifies each vertebral level, and displays two/three-dimensional planning images on the user interface. The target vertebra is manually selected and the software suggests an optimal transpedicular approach which may be adjusted in multiple planes (*e.g.,* intercostovertebral access for thoracic levels). Once the trajectory is validated, the C-arm automatically rotates to the ‘bulls-eye view’ along the planned insertional axis. The ‘virtual’ trajectory is then superimposed over the ‘real-world’ images from cameras integrated in the flat-panel detector of a standard C-arm fluoroscopy machine, and the monitor displays live video output from the four cameras (including ‘bulls-eye’ and ‘sagittal’/perpendicular views) with overlaid, motion-compensated needle trajectories. The metrics studied included trocar placement accuracy, complications, trocar deployment time, and fluoroscopy time. All procedures in both groups were successful with no complications observed in either group. No statistically significant differences in accuracy were observed between the groups. Fluoroscopy time was lower in the AI-guided group, whilst deployment time was lower in the standard-fluoroscopy group.

## Robotics

Robotic assistance is becoming essential in surgery, increasing precision and accuracy as well as the operator’s degrees of freedom compared to human ability alone. Its increased use is inevitable in IR where robotic assistance with remote control also allows for radiation protection during interventional procedures.

The majority of robotic systems currently employed in clinical practice are primarily teleoperators or assistants for tasks involving holding and precise aiming. The advancement of systems capable of operating at higher autonomy levels, especially in challenging conditions, presents considerable research hurdles. A critical aspect for such systems is their ability to consistently track surgical and IR tools and relevant anatomical structures throughout interventional procedures, accounting for organ movement and breathing. The application of deep artificial neural networks to robotic systems helps in handling multimodal data generated in robotic sensing applications [36].

Fagogenis et al. [37] demonstrated autonomous catheter navigation in the cardiovascular system using endoscopic sensors located at the catheter tip to perform paravalvular leak closure. Beating-heart navigation is particularly challenging because the blood is opaque and the cardiac tissue is moving. The endovascular endoscope acts as a combined contact and imaging sensor. ML processes camera input data from the sensor providing clear images of whatever the catheter tip is touching whilst also inferring what it is touching (*e.g.,* the blood, tissue, and valve) and how hard it is pressing.

In an article published in Nature Machine Intelligence, Chen et al. [38] described a DL driven robotic guidance system for obtaining vascular access. AI based on a recurrent fully convolutional network—Rec-FCN takes bimodal near-infrared and duplex US imaging sequences as its inputs and performs a series of complex vision tasks, including vessel segmentation, classification, and depth estimation. A three-dimensional map of the arm surface and vasculature is derived, from which the operator may select a target vessel that is subsequently tracked in real time in the presence of arm motion.

## Touchless software interaction

IR is highly technology dependent and IR suites rank amongst the most technologically advanced operating rooms in medicine. The interventionalist must interact with various hardware during procedures within the confines of a sterile environment. Furthermore, in some cases, this necessitates verbal communication with the circulating nurse or technician for the manipulation of computers in the room. Touchless software interaction could simplify and speed up direct interaction with computers, eliminating the need for an intermediary and thereby enhancing efficiency. For instance, one study utilised a voice recognition interface to adjust various parameters during laparoscopic surgery such as the initial setup of the light sources and the camera, as well as procedural steps such as the activation of the insufflator [39].

AI has emerged as an important approach to streamline these touchless software-assisted interactions using voice and gesture commands. ML frameworks can be trained to classify voice commands and gestures that physicians may employ during a procedure. These methods can contribute to improved recognition rates of these actions by cameras, speakers, and other touchless devices [40, 41].

In a study by Schwarz et al. [42], body gestures were learned by a ML software using inertial sensors worn on the head and body of the operator with a recognition rate of 90%. Body sensors eliminate issues associated with cameras such as ensuring adequate illumination or the need to perform gestures in the camera’s line of sight.

## Virtual biopsy

Virtual biopsy refers to the application of radiomics for the extraction of quantitative information not accessible through visual inspection from radiological images for tissue characterisation [66, 67].

Features from radiological images can be fed into AI models in order to derive lesions’ pathological characteristics and molecular status. Barros et al. [43] developed an AI model for digital mammography that achieved an area under the receiver operating characteristic curve of 0.76 (95% confidence interval 0.72–0.83), 0.85 (0.82–0.89), and 0.82 (0.77–0.87) for the pathologic classification of ductal carcinoma *in situ*, invasive carcinomas, and benign lesions, respectively.

In the future, virtual biopsy could partially substitute traditional biopsy, avoiding biopsy complications or providing additional information to that obtained by biopsy, especially in core biopsies where only a small amount of tissue is taken from lesions that may be very heterogeneous. However, virtual biopsy has the disadvantage of having low spatial and contrast resolution, with respect to tissue biopsy that is able to explore processes at a subcellular level.

## Conclusions

AI opens the door to a multitude of major improvements in every step of the interventional radiologist’s workflow and to completely new possibilities in the field. ML is flexible, as it learns to work for virtually any kind of application.

The evolution of AI in IR is anticipated to drive precision medicine to new heights. Tailoring treatment plans to individual patient profiles by leveraging AI-based predictive analytics could lead to more accurate diagnoses and optimised procedural strategies. The prospect of dynamic adaptation to procedural variations is on the horizon, potentially revolutionising treatment customisation in real-time.

AI-driven enhanced imaging capabilities, coupled with advanced navigational aids, are set to provide interventional radiologists with unprecedented accuracy and real-time guidance during complex procedures. Furthermore, the integration of AI with robotics is a compelling avenue, potentially steering IR towards more autonomous procedures.

The applications currently explored in medical literature just give a clue of what will be the real scenario in the future of IR. Nevertheless, AI research in IR is nascent and will encounter many technical and ethical problems, similar to those faced in diagnostic radiology. Collaborative initiatives amongst healthcare institutions to pool standardised anonymised data and promote the sharing of diverse datasets must be encouraged. Federated learning is a ML approach where models are trained collaboratively across decentralised devices without sharing raw data. It enables collaborative model development whilst preserving patient privacy [68].

Expectations are high, probably beyond the capabilities of current AI tools. A lot of work has to be done to see AI in the IR suite, with patient care improvement always as the primary goal.

## Abbreviations

AI: Artificial intelligence
AR: Augmented reality
CNN: Convolutional neural network
CT: Computed tomography
DL: Deep learning
IR: Interventional radiology
ML: Machine learning
MRI: Magnetic resonance imaging
ROI: Region of interest
TACE: Transarterial chemoembolisation
TICI: Thrombolysis in cerebral infarction
US: Ultrasound
